# Opposite Roles of RNase and Kinase Activities of Inositol-Requiring Enzyme 1 (IRE1) on HSV-1 Replication

**DOI:** 10.3390/v9090235

**Published:** 2017-08-23

**Authors:** Airong Su, Huanru Wang, Yanlei Li, Xiaohui Wang, Deyan Chen, Zhiwei Wu

**Affiliations:** 1 Center for Public Health Research, Medical School, Nanjing University, Nanjing 210093, China; suairong-1@163.com (A.S.); wanghuanru622@163.com (H.W.); sk1lyl2007@163.com (Y.L.); nlrongruo@hotmail.com (X.W.); chendeyan1234@sina.cn (D.C.); 2 State Key Lab of Analytical Chemistry for Life Science, Nanjing University, Nanjing 210023, China; 3 Jiangsu Laboratory for Molecular Medicines, Nanjing University, Nanjing 210093, China

**Keywords:** endoplasmic reticulum (ER), herpes simplex virus 1 (HSV-1), inositol-requiring enzyme 1 (IRE1), unfolded protein response (UPR), X-box binding protein 1 (XBP1)

## Abstract

In response to the endoplasmic reticulum (ER) stress induced by herpes simplex virus type 1 (HSV-1) infection, host cells activate the unfolded protein response (UPR) to reduce the protein-folding burden in the ER. The regulation of UPR upon HSV-1 infection is complex, and the downstream effectors can be detrimental to viral replication. Therefore, HSV-1 copes with the UPR to create a beneficial environment for its replication. UPR has three branches, including protein kinase RNA (PKR)-like ER kinase (PERK), inositol-requiring enzyme 1 (IRE1), and activated transcription factor 6 (ATF6). IRE1α is the most conserved branch of UPR which has both RNase and kinase activities. Previous studies have shown that IRE1α RNase activity was inactivated during HSV-1 infection. However, the effect of the two activities of IRE1α on HSV-1 replication remains unknown. Results in this study showed that IRE1α expression was up-regulated during HSV-1 infection. We found that in HEC-1-A cells, increasing RNase activity, or inhibiting kinase activity of IRE1α led to viral suppression, indicating that the kinase activity of IRE1α was beneficial, while the RNase activity was detrimental to viral replication. Further evidence showed that the kinase activity of IRE1α leads to the activation of the JNK (c-Jun N-terminal kinases) pathway, which enhances viral replication. Taken together, our evidence suggests that IRE1α is involved in HSV-1 replication, and its RNase and kinase activities play differential roles during viral infection.

## 1. Introduction

Herpes simplex virus type 1 (HSV-1), a member of the *Herpesviridae* family, is one of the most prevalent human pathogens. HSV establishes latent infection in neuronal cell bodies, and becomes reactivated when triggered by environmental or physiological factors [1,2]. The replication of herpes virus is temporally regulated by immediate early (IE), early and late genes. Although the clinical manifestations by viral infection are generally benign and self-limiting, severe or even life-threatening cases such as herpes encephalitis (HSE) are observed occasionally. There are no effective drugs that can completely eradicate the virus [3]; therefore, studies on the interaction between the virus and its host are necessary, and may shed light on antiviral strategy.

Endoplasmic reticulum (ER) is a membranous network of branching tubules and flattened sacs, running through the cytoplasm of all eukaryotic cells, and continues with the nuclear envelope. Approximately one-third of the total cellular proteins are translocated into the lumen of the ER for post-translational modification and correct folding [4]. Misfolded or unfolded proteins are transported out of the ER and degraded through ER-associated degradation (ERAD). When a large amount of newly synthesized proteins are transported into ER and are beyond the folding capability of ER, ER stress occurs. ER stress is an evolutionarily conserved cellular stress condition, which plays important roles in diverse biological processes, including metabolism, tumorigenesis and viral infection [5,6,7,8]. ER stress response can transmit stress signal from ER to nucleus by triggering signal transductions known as the unfolded protein response (UPR). UPR consists of three independent ER membrane transducers, including protein kinase RNA (PKR)-like ER kinase (PERK), activated transcription factor 6 (ATF6), and inositol-requiring enzyme 1 (IRE1). Triggering of these transducers could lead to the modulation of protein translation, protein folding, and degradation to either reestablish the homeostasis or activate apoptosis when the stress persists [9,10]. Virus infections could induce UPR by accumulating unfolded or misfolded proteins in the ER lumen when a burst of viral protein synthesis occurs. On the other hand, UPR was shown to influence the replication of a number of viruses including hepatitis C virus (HCV) [11], Japanese encephalitis virus (JEV) [12] and HSV-1 [13], etc.

The IRE1α/XBP1 (X-box binding protein 1) branch is the most conserved signaling pathway in UPR from yeast to humans. IRE1 has two isoforms in mammalian cells: IRE1α and IRE1β. IRE1α is expressed in most tissues and cells, while the expression of IRE1β is primarily restricted to intestinal epithelial cells [14]. IRE1α possesses both kinase and endoribonuclease (RNase) activity at its C-terminal cytosolic moiety [15], and its N-terminal luminal domain acts as a sensor of unfolded or misfolded proteins under stress conditions [16]. BiP (binding immunoglobulin protein), also known as glucose-regulated protein 78 (GRP78), constitutively binds to IRE1α to prevent its activation under nonstress conditions. In response to ER stress, BiP binds to the unfolded proteins and releases from IRE1α, leading to IRE1α activation [17]. XBP1 mRNA is the first discovered substrate for the IRE1α RNase. The activation of IRE1α results in its dimerization and autophosphorylation, leading to the cleavage of XBP1 mRNA via an unconventional splicing reaction [18]. Expression of the spliced XBP1 (XBP1s) acts as a transcription factor for the expression of genes which are critical for efficient protein folding, maturation, and degradation [19]. Besides of XBP1 pre-mRNA, RNase activity of IRE1α also mediates the degradation of a select subset of cellular mRNAs, that is termed regulated IRE1-dependent decay (RIDD) [20]. RIDD cleaved pre-mRNAs at a site different from the XBP1 spliced site, and either maintains ER homeostasis or induces apoptosis [21]. If the ER stress is not mitigated, the activated IRE1α can also lead to the activation of JNKs (c-Jun N-terminal kinases) by recruiting and clustering the TNF receptor-associated factor 2 (TRAF2), and then triggers apoptosis [22]. 

IRE1α was reported to be involved in many viral infections. Hassan et al. reported that influenza A viral replication activated the IRE1 branch of UPR [23]. The IRE1α pathway is also activated during a murine coronavirus mouse hepatitis virus (MHV) infection [24]. Another study observed that hepatitis C virus suppressed the IRE1α-XBP1 pathway during infection [25]. Human cytomegalovirus (HCMV) is a betaherpes virus, which can also inhibit the XBP1 target gene *EDEM* (ER degradation enhancing α-mannosidase-like protein) to benefit viral replication [26]. The investigation of cellular UPR during HSV-1 replication has been mainly focused on the PERK signaling pathway [13,27,28]. Although studies have shown that RNase activity of IRE1α was inactivated during HSV-1 infection [29,30], it remains unknown about the effect of IRE1α RNase activity on viral replication. Stress-induced JNKs activation was reported to be important for the efficiency of HSV-1 viral replication since JNK inhibitors could reduce the viral yield by 70% [31,32]. Since activated IRE1α can lead to JNKs activation [22], the relationship between JNKs and IRE1α during viral infection deserves further investigation. In the current study, we found that the IRE1α expression was up-regulated during HSV-1 infection, which suggests that the IRE1α branch may be involved in the regulation of HSV-1 replication. Further study showed that when IRE1α RNase activity was increased by either pretreating cells with chemical compounds or XBP1s over-expression, the HSV-1 replication was repressed, suggesting that the RNase activity of IRE1α is unfavorable for the virus. We presented evidence that the JNK is activated by IRE1α kinase activity, and thus facilitates viral replication. In conclusion, our research indicates that HSV-1 replication is regulated by both the RNase and the kinase activities of IRE1α, and these two activities have distinct effects on viral replication.

## 2. Materials and Methods

### 2.1. Reagents, Cell Lines and Viruses

IRDye 680 goat-anti-rabbit and IRDye 800 goat-anti-mouse were obtained from LI-COR (Lincoln, NE, USA). Antibodies specific for Glycoprotein D (gD-1), glyceraldehyde 3-phosphate dehydrogenase (GAPDH), Jun N-terminal protein kinase 2 (JNK2), and radio immunoprecipitation assay (RIPA) lysis buffer were purchased from Santa Cruz Biotechnology (Santa Cruz, CA, USA). Antibodies specific for IRE1α and phophorylated Jun N-terminal protein kinase 1 and 2 (p-JNK1/2) were purchased from Cell Signaling Technology (Beverly, MA, USA). Anti-IRE1α (phospho S724) was purchased from Abcam (Cambridge, MA, USA). DRAQ5 was obtained from eBioscience (San Diego, CA, USA). Thapsigargin (Tg) and phosphonoacetic acid (PAA) were purchased from Sigma-Aldrich (St. Louis, MO, USA). APY29 and STF-083010 were obtained from MedChemExpresss (MCE) (Princeton, NJ, USA). SP600125 was purchased from Beyotime (Haimen, Jiangsu, China). Plasmid encoding XBP1s was cloned into p3×FLAG-CMV-7.1 expression vector (St. Louis, MO, USA). Anti-FLAGM2 antibody was purchased from Sigma-Aldrich. IRE1 alpha KA-pcDNA3.EGFP and IRE1 alpha-pcDNA3.EGFP were gifts from Fumihiko Urano (Addgene plasmid # 13010, Addgene plasmid # 13009).

Hela, Vero, and HEC-1-A cells were obtained from American Type Culture Collection (ATCC, Manassas, VA, USA). The cells were cultured in Dulbecco’s Modified Eagle’s medium (DMEM) or McCoy’s 5A, supplemented with 10% fetal bovine serum (Life Technologies, Carlsbad, CA, USA). HSV-1 (strain HF), a attenuated strain of the virus, originated from ATCC^®^ VR260™ [33], was widely used in the researches of either antiviral study or the relationship between host cells and virus [34,35,36]. HSV-1 (HF) was propagated and titrated on Vero cells as described previously [37].

### 2.2. In Vitro Antiviral Assay

The in vitro antiviral activity of APY29, Tg, and STF-083010 were determined by In-cell Western assay, or by titrating the infectious virion in drugs-treated cells as described [34]. Briefly, 2 × 10^4^ cells were dispersed into each well of 96-well plates and, after 24 h culture, the cells were either mock-pretreated or pretreated with drug for 30 min at 37 °C, and then infected with HSV-1 (HF) (multiplicity of infection (moi) = 1) by adding the virus directly into the culture medium. Viral protein expression was determined by In-cell Western analysis of gD-1 expression as described below. 

Viral titration analysis was evaluated as follows: confluent HEC-1-A cells cultured in a 96-well plate were pretreated with serial concentrations of drug for 30 min and then infected with HSV-1 (moi = 1) for 24 h, and the cultural medium was replaced with 200 μL fresh medium. HSV-1-infected cells were frozen and then thawed with three cycles to release the virion. The virion-containing medium was diluted and dispensed on confluent Vero cell monolayers. After 72 h of incubation, viral titration was performed by counting the numbers of plaque-forming units (PFU).

### 2.3. Western Blot and In-Cell Western

Cells were lysed using RIPA lysis buffer on ice for 30 min and then centrifuged at 12,000× *g* for 10 min at 4 °C to remove insoluble debris. The supernatants were collected and total protein concentrations were determined using bicinchoninic acid assay (BCA) protein assay kit (Pierce, Rockford, IL, USA). After boiling at 95 °C for 5 min, the proteins were separated on 10% SDS-PAGE with reducing condition and transferred to polyvinylidene difluoride (PVDF) membranes (Millipore, Billerica, MA, USA). The membranes were blocked using Odyssey Blocking buffer (LI-COR) and then stained with primary antibodies for 2 h at room temperature, followed by washing with phosphate-buffered saline (PBS)-0.1% tween-20 (PBS-T buffer) 5 min for 5 times and staining with Infrared dye (IRDye) IgG (1:10,000) for 1 h at RT. The proteins were visualized under LI-COR Odyssey Infrared Imager (LI-COR), after washing with PBS-0.1% tween-20 (PBS-T buffer) 5 min for 5 times.

In-cell Western assay was performed in 96-well plate. The cells were fixed with 4% paraformaldehyde for 20 min at room temperature, and permeabilized by 5 washes of 0.1% Triton-X 100 in PBS with 5 min for each wash. Cell monolayers were blocked using Odyssey Blocking buffer (LI-COR) for 90 min and then incubated with primary antibodies being diluted in blocking buffer (1:200) for 2 h at RT. After being washed with PBS-T buffer, the monolayers were stained in IRDye IgG (1:1500) for 1 h. The plate was rinsed and scanned in Odyssey Infrared Imager. Relative protein expression was normalized against DRAQ5 fluorescence and quantified.

### 2.4. RNA Extraction and Real-Time RT-PCR Analysis

Total RNA was extracted using TRIzol reagent (Life Technologies), according to the manufacturer’s protocol. Equivalent amounts of RNA (1 μg) from each sample were subjected to reverse-transcription using ReverTra Ace qPCR RT kit (TOYOBO, Osaka, Japan). Real-time PCR was performed in triplicate on ABI Prism 7300 Sequence Detection System using SYBR Green PCR Master Mix (Life Technologies). We determined the mRNA level of spliced XBP1 by real-time PCR using a primer set that selectively amplifies the spliced variant of XBP1 cDNA. The forward primer used in this reaction is designed to span the 26-base pair (bp) intron, so only the spliced XBP1 cDNA was amplified [38]. Primers of *XBP1s*, *IRE1α*, *EDEM*, *Herp*, and *gD* used in real-time PCR are: *XBP1s* forward: 5′-GGTCTGCTGAGTCCGCAGCAGG-3′, and reverse: 5′-GGGCTTGGTATATATGTGG-3′, *IRE1α* forward: 5′-CGGCCTTTGCAGATAGTCTC-3′, and reverse: 5′-ACGTCCCCAGATTCACTG-3′, *EDEM* forward: 5′-GCACAGGCCGAAACCTCAT-3′, and reverse: 5′-TGCTCTTTAAGGGCAGGGAG-3′, *Herp* forward: 5′-CACCGCGACTTGGAGCTGAGTGG-3′, and reverse: 5′-TCTGTGGATTCAGCCACCTTGG-3′, *gD* forward: 5′-AGCAGGGGTTAGGGAGTTG-3′, and reverse: 5′-CCATCTTGAGAGAGGCATC-3′. Messenger RNA transcription levels were standardized against housekeeping gene *GAPDH* (forward, 5′-TGCACCACCAACTGCTTAGC-3′, and reverse, 5′-GGCATGGACTGTGGTCATGAG-3′).

### 2.5. XBP1 mRNA Splicing Assay

To measure the degree of *XBP1* mRNA splicing, the total cellular RNA was isolated as described above. XBP1 was PCR amplified using Platinum^TM^ Hot Start PCR Master Mix (Life Technologies), according to the manufacturer′s instructions. In order to amplify both the spliced and the unspliced variants of XBP1 cDNA, the primers were designed circumventing the spliced segment as follows: forward primer 5′-CCTTGTAGTTGAGAACCAGG-3′, and reverse primer, 5′-GGGCTTGGTATATATGTGG-3′. For the analysis of PCR products, 10 μL of each reaction mixture was loaded on a 2.5% agarose gel in TBE (45 mM Tris-borate, 1 mM EDTA, pH 8.0), and subjected to electrophoresis to separate the products. The bands were visualized using Gel Imaging System (Tianneng 3500, Shanghai, China).

### 2.6. Small Interfering RNA (siRNA) Analysis

To determine the effect of IRE1α kinase activity on HSV-1 replication, the IRE1α expression was knocked-down using X-treme GENE siRNA Transfection Reagent (Sigma-Aldrich, St. Louis, MO, USA), according to the manufacturer’s instructions. Hela cells in 6-well plates were transfected with the target siRNA (1.5 μg/well) or control siRNA. The *IRE1α* siRNA sequence is: GGACGUGAGCGACAGAAUAdTdT, for negative control (NC): UUCUCCGAACGUGUCACGUTT. siRNAs were synthesized by GenePharma (Shanghai, China). Cells were mock-infected or infected with HSV-1 (HF) (moi = 1) at 48 h post transfection, harvested at 24 h post-infection (p.i.), and samples were prepared for translation and transcription expression analysis using Western blot or real-time PCR methods. 

### 2.7. Cell Transfection and HSV-1/Blue Assay

In order to examine the effect of XBP1s on viral infection, Hela cells were transiently transfected with plasmid cloning vehicle pcDNA3.1 or XBP1s plasmid using Lipofectamine 3000 transfection reagent (Life Technologies). 2.5 μg/well of plasmid was used to transfect Hela cells in 6-well plate, 100 ng/well of plasmid was used to transfect cells in 96-well plate. After incubation for 48 h, the cells were infected with HSV-1/blue (moi = 1) for 24 h (cultured in 6-well plate) or 12 h (cultured in 96-well plate), before being lysed with RIPA lysis buffer or 1% NP-40 in PBS. The cell lysates in 6-well plate were harvested and preparing for Western blot analysis, and in 96-well plate were then transferred to a new Costar 96-well flat plate and mixed with CPRG (chlorophenol red-β-d-galactopyranoside; Boehringer, Ingelheim, Germany), and β-gal activity was measured in a TECAN Infinit M200 microplate reader at 570 nm after 1 h.

To further investigate the effect of the kinase activity of IRE1 on viral replication, Hela cells cultured in a 6-well plate were transiently transfected with plasmid cloning vehicle pcDNA 3.1, or IRE1 alpha KA-pcDNA3.EGFP, or IRE1 alpha-pcDNA3.EGFP plasmid (2.5 μg/well) using Lipofectamine 3000 transfection reagent (Life Technologies), according to the manufacturer’s instructions. The cells were further cultured for 48 h and then mock-infected or infected with HSV-1 (HF) (moi = 1). Total cellular protein was extracted after 24 h p.i. preparing for Western blot analysis with antibodies against total IRE1α, p-IRE1α and gD, respectively.

### 2.8. In Vitro Cytotoxicity Assay

The in vitro cytotoxicity of Tg, APY29 and STF-083010 was measured using a commercial CCK-8 kit (Dojindo, Kumamoto, Japan) via colorimetric method according to the manufacturer’s instructions (see Supplementary section).

### 2.9. Statistics

Statistical analysis was performed using the two-tailed student’s *t*-test, by using SPSS 18.0 (SPSS for Windows Release 18.0, SPSS Inc., Chicago, IL, USA). Statistical significance: * *p* < 0.05, ** *p* < 0.01.

## 3. Results

### 3.1. IRE1α Expression Was Up-Regulated during HSV-1 Infection

**Figure 1:**
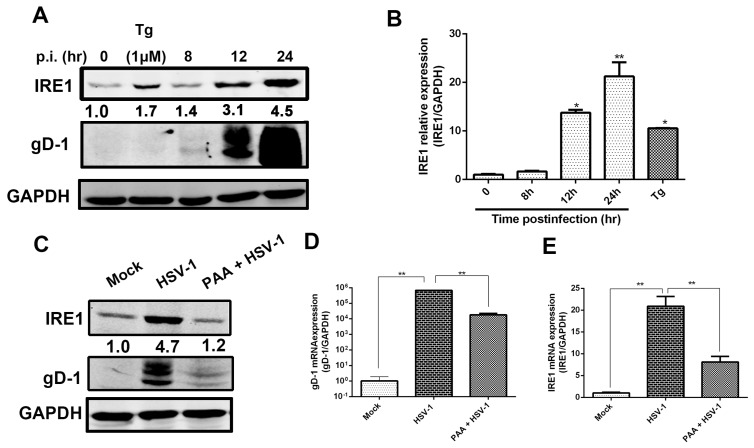
Inositol-requiring enzyme 1 (IRE1α) expression is up-regulated by herpes simplex virus type 1 (HSV-1) infection. (**A**,**B**) HSV-1 infection increased IRE1α expression in HEC-1-A cells. HEC-1-A cells were infected with HSV-1 (strain HF) at multiplicity of infection (moi) = 1. (**A**) Total cellular protein was harvested at indicated time point p.i. (post infection) and subject to Western blot analysis using antibodies against total IRE1, gD-1 (Glycoprotein D) and GAPDH (glyceraldehyde 3-phosphate dehydrogenase). The number under each band represents the relative density of the band in comparison to the corresponding control normalized to GAPDH. Cells treated with Thapsigargin (Tg) (1 μM) harvested at 4 h was used as a positive control; (**B**) IRE1α transcription expression was determined by real-time PCR at various time points post-infection (p.i.). Total RNA was extracted, IRE1 mRNA level was measured by real-time PCR and normalized to GAPDH. Cells treated with Tg (1 μM) for 4 h was served as control. The data are presented as mean ± standard deviation (SD) from three independent determinations (* *p* < 0.05, ** *p* < 0.01); (**C**–**E**) Both IRE1α and gD expression were inhibited by PAA. HEC-1-A cells were pretreated or mock-pretreated with phosphonoacetic acid (PAA, 400 μg/mL) for 30 min and then infected or mock-infected with HSV-1 (HF) at moi = 1. Both protein and mRNA transcription levels of IRE1α and gD-1 at 24 h p.i. were determined. Columns and bars represent the mean ± SD of three independent experiments (** *p* < 0.01). All experiments were performed three times. Representative results are shown.

Both protein and mRNA levels of IRE1α were analyzed to illustrate the effect of viral infection on IRE1α stress pathway after cells were infected with HSV-1 (HF) (moi = 1). Cells pretreated with Tg, an ER stress inducer which induces strong IRE1 RNase activity [7,30], served as a positive control. The results showed that the IRE1α protein expression was markedly up-regulated by HSV-1 infection from 12 h p.i. (Figure 1A). 

The mRNA level of IRE1α was also up-regulated correspondingly (Figure 1B). We further investigated whether the up-regulations of IRE1α protein and mRNA levels were due to viral DNA replication. Before being infected with HSV-1 (moi = 1), HEC-1-A cells were pretreated with PAA (400 μg/mL), an inhibitor of viral DNA polymerase, and viral replication [13]. Both the mRNA and protein levels of gD and IRE1α were determined by either real-time PCR or Western blot analysis. The results showed that PAA could efficiently reduce the translation and replication of HSV-1, as indicated by the reduced gD protein and mRNA expressions. Both the protein and the mRNA levels of IRE1α were also significantly reduced (Figure 1C–E), indicating that the cellular IRE1α signaling pathway is likely to be affected by HSV-1 replication.

### 3.2. HSV-1 Replication Was Inhibited by the RNase Activity of IRE1α

**Figure 2:**
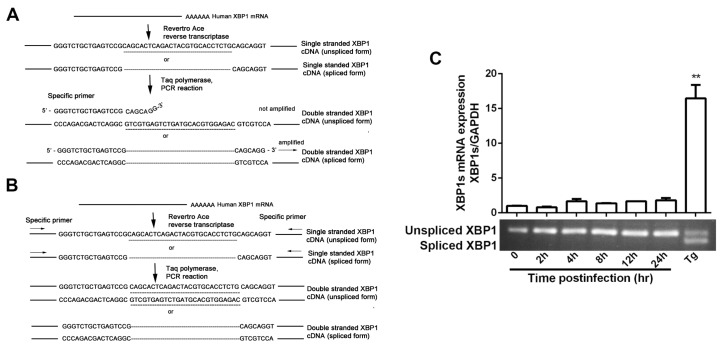

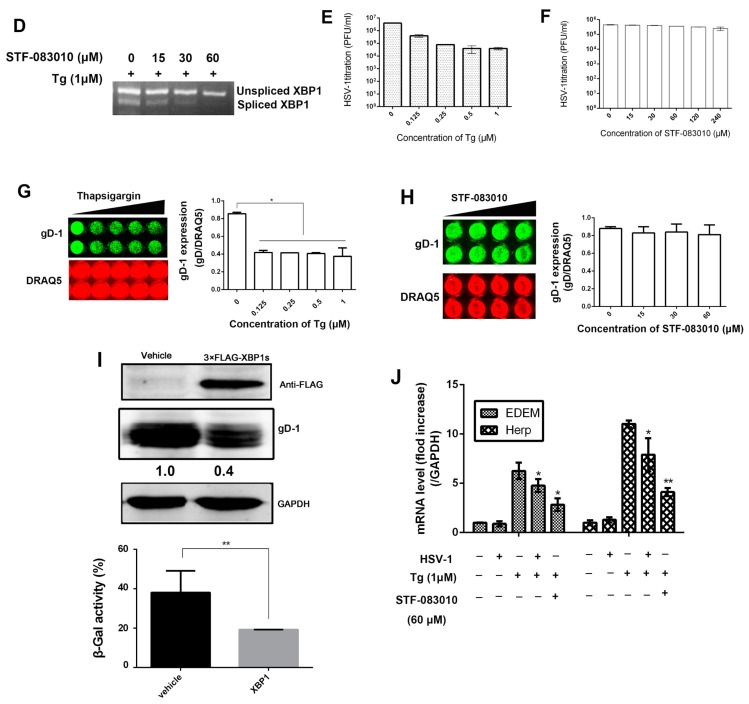
HSV-1 viral replication was inhibited by RNase activity of IRE1α. (**A**) The PCR approach for both unspliced X-box binding protein 1 (*XBP1*) and spliced *XBP1* genes. Total RNA was extracted and reverse-transcribed, spliced *XBP1* mRNA was amplified by real-time PCR using the specific primers [38]. The underlined nucleotides show the 26 bp intron sequence that is spliced out when ER stress occurred; (**B**) The double-stranded cDNA was synthesized by PCR using specific sense and anti-sense primers for both the unspliced and spliced *XBP1* genes [38]; (**C**) The effect of viral infection on the spliced XBP1 (XBP1s) mRNA expression. HEC-1-A cells were infected with HSV-1 (HF) (moi = 1), total cellular RNA was extracted at the indicated times and the XBP1s mRNA expression was determined by real-time PCR (the upper part of the panel) and nucleic acid electrophoresis (the lower band) using the primers set as shown in the text. Cells were treated with Tg (1 μM) for 4 h served as control; (**D**) Spliced and unspliced *XBP1* products of PCR amplification were showed by nucleic acid electrophoresis; (**E**,**F**) Consequence of Tg or STF-083010 treatment on HSV-1 replication. HEC-1-A cells were pretreated with serial concentrations of Tg or STF-083010 for 30 min and then infected with HSV-1 (HF) (moi = 1) for 24 h. The infectious viral particles from infected cells were titrated by measuring the plaque forming unit (PFU); (**G**,**H**) The effect of Tg or STF-083010 on HSV-1 gD expression. HEC-1-A cells were pretreated with serial concentrations of Tg or STF-083010 for 30 min and then infected with HSV-1 (HF) (moi = 1), gD-1 protein expression in the presence of drugs was determined via In-cell Western and normalized against DRAQ5 fluorescence at 24 h p.i.; (**I**) Effect of over-expression of XBP1s on HSV-1 replication. Vehicle pcDNA 3.1 or 3 ×FLAG-XBP1s plasmid was transfected into Hela cells for 48 h before cells were infected with HSV-1 (HF) (moi = 1). The cells were lysed after 24 h p.i. then XBP1s and gD-1 expressions were determined by Western blot analysis using antibodies against anti-FLAG and gD (the upper panel). Hela cells were infected with HSV-1/blue (moi = 1) after vehicle or 3 ×FLAG-XBP1s plasmid was transfected into cells for 48 h. The β-Gal activity was measured as described in the text 12 h p.i. (the lower panel); (**J**) The transcription levels of both *EDEM* and *Herp* were downregulated by HSV-1 infection. HEC-1-A cells were pretreated or mock-pretreated with Tg for 30 min then mock-infected or infected with HSV-1 (moi = 1) or treated with STF-083010 (60 μM). Total cellular RNA was extracted after 12 h p.i. and the mRNA levels were determined by real-time PCR. All experiments were performed three times. The representative results were shown. Data are mean values (±SD) of three independent experiments (* *p* < 0.05, ** *p* < 0.01), Error bars show standard deviations (SD) from three separate experiments.

The IRE1α RNase activation can induce the splicing of XBP1 mRNA [39]. The translation product (XBP1s) of the spliced XBP1 mRNA is an important transcriptional regulator of genes involved in protein folding and degradation [40,41]. To investigate if IRE1α RNase activity was also affected by HSV-1 infection, we determined the spliced XBP1 mRNA level by real-time PCR by using a primer set that selectively amplifies the spliced variant of XBP1 cDNA [38], and the degree of XBP1 splicing using primers that circumvent the spliced segment, which allows for both the spliced and the unspliced variants of XBP1 cDNA to be amplified (Figure 2A,B). Tg is an effective inhibitor of the Ca^2+^ ion pump proteins of intracellular membranes, located in sarcoplasmic reticulum (SR) and endoplasmic reticulum (ER) [42]. Tg is widely used as an inducer of ER stress [7,30].

The results showed that the *XBP1s* mRNA level and the *XBP1* splicing degree were constant during viral infection, but increased in Tg-treated cells (Figure 2C), which is consistent with previous reports [29,30], and confirms that IRE1α RNase activity was not activated by HSV-1 infection.

XBP1s plays a vital role in the regulation of a large number of genes functional in ERAD which may negatively regulate viral replication [30,43,44]. Previous study showed that HCV suppressed the IRE1-XBP1 pathway to facilitate its replication [25]. Recent research found that the IRE1-XBP1 pathway was also inhibited by HSV-1 UL41 protein [30], but the effect of IRE1α RNase activity on HSV-1 remains uncertain. Therefore, we investigated the effect of IRE1α RNase activity on HSV-1 replication. STF-083010, an inhibitor of IRE1α RNase activity [45] that suppressed the Tg-induced XBP1 splicing in a dose dependent manner (Figure 2D), was used as a means for demonstrating the effect of reducing RNase activity of IRE1α on HSV-1 infection. Tg and STF-083010, which have opposite effects on XBP1 splicing, were used to treat cells before viral infection, and their effects on the viral infection were evaluated by measuring the PFU 24 h p.i. Tg but not STF-083010 inhibited the replication of HSV-1, as shown in Figure 2E,F. The inhibitory effect of Tg on viral replication was supported by gD reduction in the presence of the drug, as shown in Figure 2G. Consistently, STF-083010 showed no effect on gD expression (Figure 2H). Since the cell viability was not affected by the drug at the experimental concentrations as determined by CCK-8 colorimetric assay, we ruled out the possibility that the viral inhibitory activity was due to the cytotoxic effect of the drug (the data was shown in the supplementary material).

To further illustrate the role of XBP1s on viral replication, we overexpressed XBP1s by plasmid transfection, followed by the infection of the cells with HSV-1. gD protein level was measured by Western blot, and the result showed that the gD expression was reduced by about 60% in the cells overexpressing XBP1s, indicating that viral replication was suppressed by XBP1s (upper panel of Figure 2I). We also employed an HSV-1/blue recombinant virus assay to confirm the effect of spliced XBP1 on viral replication. This recombinant virus contains an HSV-1 ICP4 promoter-driven lacZ gene, inserted into the HSV-1 TK gene loci. We determined the effect of XBP1s overexpression on viral replication by measuring the regulated expression of β-galactosidase of the recombinant virus as a marker for viral growth [46,47]. The result showed that the replication of HSV-1/blue was reduced, indicating that ICP4 promoter activity was inhibited in the Hela cells overexpressing XBP1s (lower panel of Figure 2I). During the ER stress responses, terminally unfolded or misfolded proteins may be degraded by ERAD. We speculated that the viral proteins would be degraded by ERAD when a large amount of viral proteins accumulated in ER during replication. HSV-1 may have evolved countermeasures to IRE1-XBP1 action to prevent detrimental effect induced by XBP1 splicing, and this speculation was supported by a previous study which showed that XBP1 splicing induced by Tg was repressed by HSV-1 UL41 protein [30]. To explore if the downstream of XBP1 was also inhibited by HSV-1 infection, we measured the transcriptions of both *EDEM* and *Herp* genes, both of which are regulated by XBP1s and directly participate in ERAD [43,48]. HEC-1-A cells were cultured in the presence of Tg (1 μM) for 30 min and then infected with HSV-1 (moi = 1) for 12 h and the transcriptions of both *EDEM* and *Herp* were determined by real-time PCR. The results showed that increased transcriptions of these two genes induced by Tg were downregulated by HSV-1 infection (Figure 2J), suggesting that HSV-1 suppressed ERAD by inhibiting the RNase activity of IRE1α to avoid degradation of the viral proteins. STF-083010 (60 μM), used as control, significantly suppressed Tg-induced XBP1 splicing (2D), and the transcription of both *EDEM* and *Herp* (2J). Taken together, the results suggest that RNase activity of IRE1α is detrimental to the viral replication, and were not activated during viral infection.

### 3.3. The Effect of IRE1α Kinase Activity on HSV-1 Replication

**Figure 3:**
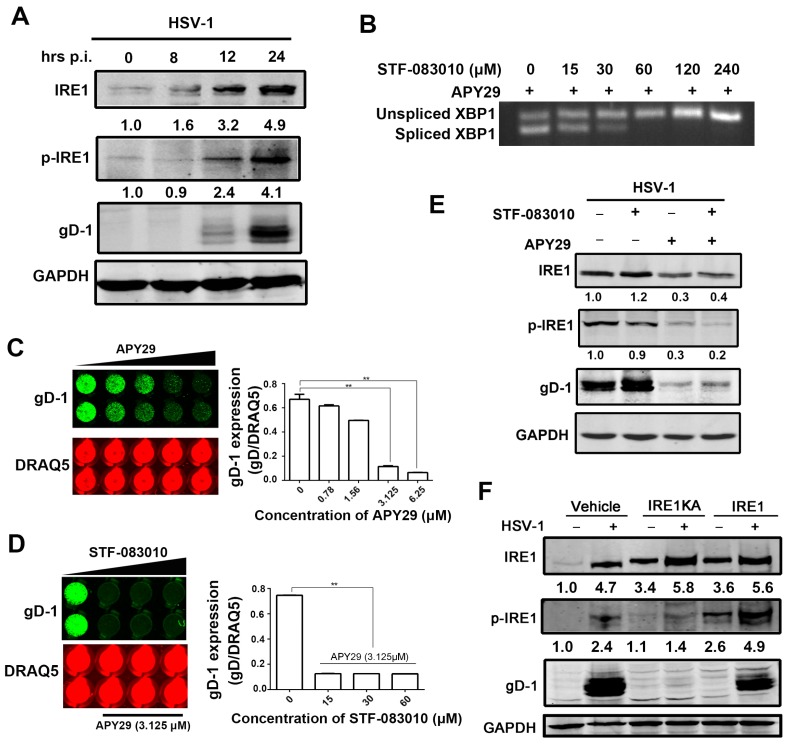
HSV-1 replication is regulated by kinase activity of IRE1α. (**A**) HSV-1 infection stimulated kinase activity of IRE1α in HEC-1-A cells. HEC-1-A cells were infected with HSV-1 (HF) (moi = 1). Cell lysates were prepared at the times indicated and subject to Western blot analysis using antibodies against total IRE1α, phosphorylated IRE1α, gD-1 and GAPDH; (**B**) XBP1 splicing induced by APY29 was attenuated by STF-083010 in a dose dependent manner. HEC-1-A cells were treated with both a serial concentration of STF-083010 and 3.125 μM of APY29, and then incubated for 24 h. Total RNA was prepared for measuring the expression of spliced and unspliced XBP1using reverse transcription-PCR analysis; (**C**,**D**) Viral replication was inhibited by APY29; (**C**) Confluent HEC-1-A cells cultured in a 96-well plate were pretreated with serial concentrations of APY29 and then infected with HSV-1 (HF) (moi = 1) for 24 h; (**D**) Before HEC-1-A cells were infected with HSV-1 (HF) (moi = 1), cells were incubated with both serial concentration of STF-083010 and 3.125 μM of APY29. gD-1 protein expression was determined via In-cell Western, and normalized by DRAQ5 fluorescence at 24 h p.i. Data are mean values ± SD of triplicate determinations (** *p* < 0.01). Representative results are shown; (**E**) Inhibition of IRE1α kinase activity had an inhibitory effect on HSV-1 replication in HEC-1-A cells. Cells were presence or absence of APY29 (3.125 μM) or STF-083010 (60 μM) or both of two drugs and then infected with HSV-1 (HF) at moi = 1. Total cellular cells lysates were prepared at 24 h p.i. and subject to Western blot analysis against antibodies to total IRE1α, phosphorylated IRE1α and gD-1; (**F**) Inactivation of IRE1α kinase activity suppressed HSV-1 replication in Hela cells. Hela cells were transfected with vehicle or IRE1 alpha KA-pcDNA3.EGFP or IRE1 alpha-pcDNA3.EGFP plasmid for 48 h and then infected or mock-infected with HSV-1 (moi = 1). The total and phosphorylated IRE1α, and gD-1 protein were determined by Western blot at 24 h p.i. All experiments were performed three times. The representative results were shown.

Next, we investigated the effect of kinase activity of IRE1α on viral replication. IRE1 phosphorylation is generally considered as a reliable indicator of IRE1α kinase activity [45,49,50,51]. Kinase activity of IRE1α was induced at a later stage of viral infection, as indicated by the phosphorylation of IRE1α from 12 h p.i. (Figure 3A).

APY29, an inhibitor of kinase activity of IRE1α, and also an activator of RNase activity of IRE1α [49,52], was used to illustrate the roles of kinase activity of IRE1α on HSV-1 replication. To exclude the impact of RNase activity of IRE1α on viral replication, we used STF-083010 to inhibit the APY29-induced RNase activity since XBP1 splicing induced by APY29 was inhibited by STF-083010 (Figure 3B). HSV-1 replication was inhibited by APY29 in a dose dependent manner, as shown by In-cell Western analysis on gD-1 expression (Figure 3C). When cells were pretreated with both APY29 and STF-083010 and then infected with HSV-1, APY29 showed an inhibitory effect on viral replication independent of the presence of STF-083010 (Figure 3D), suggests that the suppression of kinase activity of IRE1α by APY29 is sufficient to inhibit viral replication. This conclusion was supported by the result that down-regulation of phosphorylated IRE1α, induced by APY29 compared with that of the control group, gD expression was suppressed, as shown in Figure 3E. To further determine the role of kinase activity of IRE1α on viral replication, Hela cells were transfected with a kinase-inactive mutant K599A human IRE1α (IRE1KA) [53], and its effect on HSV-1 replication was analyzed by Western blot analysis. The result showed that gD expression was completely inhibited by IRE1KA, but not the wild-type of IRE1α (Figure 3F), suggesting that the kinase activity of IRE1α is required for the viral replication. 

### 3.4. Kinase Activity of IRE1α Enhanced HSV-1 Replication via Activating JNK Signal Pathway

**Figure 4:**
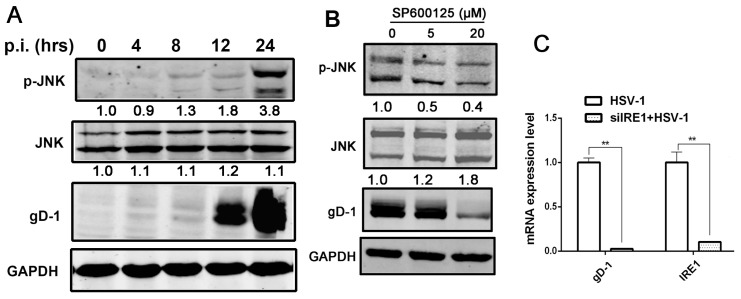

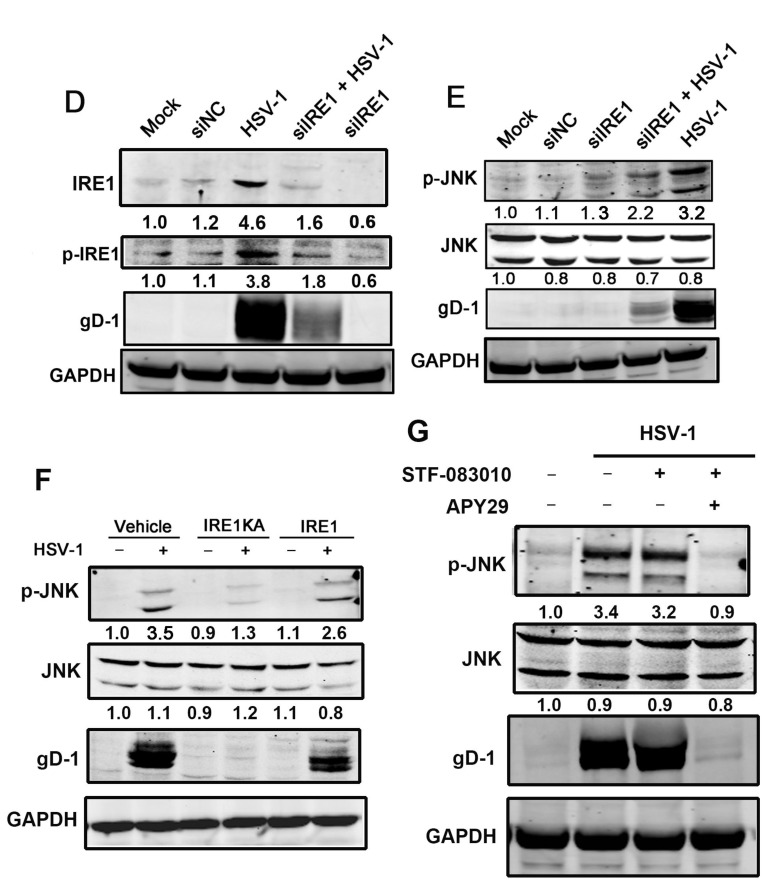
HSV-1 viral replication was regulated by kinase activity of IRE1α via JNK signal pathway. (**A**) HSV-1 infection induced the activation of JNK pathway in HEC-1-A cells. HEC-1-A cells were infected with HSV-1 (moi = 1). Whole-cell lysates were prepared from infected cells at the post infection times indicated and subject to Western blot analysis using antibodies against phosphorylated JNK, JNK, and gD. The number under each band represents the relative density of the band in comparison to the corresponding control normalized to GAPDH; (**B**) SP600125 had an inhibitory effect on viral replication in HEC-1-A cells. HEC-1-A cells were pretreated with serial concentrations of SP600125 for 30 min and then infected with HSV-1 (HF) (moi = 1). The total and phosphorylated JNK, and gD protein were analyzed by Western blot at 24 h p.i. The number under each band represents the relative density of the band in comparison to the corresponding control normalized to GAPDH; (**C**) IRE1α knock-down reduced HSV-1 viral gene replication in Hela cells. Hela cells were transfected with scramble siRNA as negative control (NC) or IRE1α siRNA for 48 h and then infected with HSV-1 (moi = 1), total RNA was extracted at 24 h p.i., both viral gD RNA level and IRE1α mRNA level were determined by real-time PCR and normalized to GAPDH. Data are means of three independent wells, and the bars represent the mean values ± SD, ** *p* < 0.01; (**D**) IRE1α knock down reduced the kinase activity of IRE1α and viral replication in Hela cells. Hela cells were treated according to material and methods Section 6, total cellular protein was harvested at 24 h p.i. and then phosphorylated IRE1α level, total IRE1α and gD expression were measured by Western blot using indicated antibodies; (**E**) IRE1α knock-down attenuated JNK activation stimulated by HSV-1 in Hela cells. Hela cells were treated according to material and methods Section 6, total cellular protein was harvested at 24 h p.i. preparing for Western blot analysis against gD, total and phosphorylated JNK antibodies; (**F**) HSV-1 stimulated JNK activation was suppressed by inactivation of IRE1α kinase activity in Hela cells. Hela cells were treated as describe in material and methods 7. Total cell lysates were prepared after 24 h p.i. for Western blot analysis using antibodies directed against gD, phosphorylated JNK and JNK; (**G**) IRE1α kinase activity inhibitor repressed JNK activation induced by viral infection in HEC-1-A cells. HEC-1-A cells were pretreated or mock-pretreated with APY29 (3.125 μM), only or both APY29 (3.125 μM), and STF-083010 (60 μM) for 30 min and then infected or mock-infected with HSV-1 (moi = 1). Total cell lysates were prepared and Western blot analysis was performed using specific antibodies at 24 h p.i. All experiments were performed three times. The representative results were shown.

Stress-induced oligomerization and activation of the IRE1α lead to clustering of TRAF2, resulting in the activation of proximal components of the JNK kinase cascade [22]. Studies showed that JNK activation could facilitate HSV replication [31]. Consistent with early observations, we showed that HSV-1 replication stimulated the JNK signaling (Figure 4A), and that the inhibition of JNK activation by an JNK inhibitor (sp600125) resulted in the suppression of viral replication (Figure 4B). 

Since JNK can be activated by IRE1α during ER stress, and the activation of JNK is important to HSV replication [31,54], we investigated if HSV-1-induced IRE1α up-regulation could activate JNK signaling pathway. In a previous study, *IRE1*^−/−^ fibroblasts were reported to be impaired in JNK activation during ER stress induced by Tg [22]. We, therefore, analyzed the effect of *IRE1α* down-regulation by RNA interference (RNAi) on JNK activation during viral infection. The results showed that compared with siNC, siIRE1α significantly down-regulated IRE1α and reduced gD expression (Figure 4C,D). Consistent with the observations, the JNK activation (p-JNK) induced by HSV-1 infection was also reduced by *IRE1α* knockdown, as shown in Figure 4E, suggesting that IRE1α pathway is one of the mechanisms mediating the JNK activation during HSV-1 infection. To further explore the mechanism of IRE1α-mediated JNK activation, we examined the effect of wild-type IRE1α and IRE1KA on JNK activation and showed that both viral replication, and JNK phosphorylation were suppressed in IRE1KA (Figure 4F). We further examined the effect of the combination of APY29 and STF-083010 on JNK signaling activation and showed that the treatment of cells with these two drugs significantly inhibited the virus-induced JNK activation, and the viral replication (Figure 4G). Taken together, the evidences suggest that the kinase activity of IRE1α is involved in the regulation of HSV-1 replication through JNK signaling pathway. We, therefore, postulate that HSV-1 replication is facilitated by the kinase activity of IRE1α while inhibited by the RNase activity of IRE1α.

## 4. Discussion

As one of the most conserved branch of UPR signaling pathways from yeast to mammals, IRE1α was reported being involved in many viral replications. Regulators of IRE1α RNase activity, Tg, and STF-083010, were reported to modulate RNase activity of IRE1α in both RIDD and XBP1 splicing processes, both of these two processes are activated by Tg and inhibited by STF-083010 [7,55]. Although RIDD was induced during ER stress, the target genes modulated by RIDD are quite complicated, and those published substrates of RIDD still lack fully validation [56], so most studies select XBP1 splicing to represent the RNase activity of IRE1α. In order to facilitate viral replication, IRE1-XBP1 pathway that mediates IRE1α RNase activity was suppressed in infection by the Hepatitis C virus, human cytomegalovirus, and Rotavirus while activated in infections of Influenza A virus, Murine coronavirus mouse hepatitis virus, Hepatitis B virus, Japanese encephalitis virus, Flavivirus, and Epstein-Barr virus [7,23,24,25,26,57,58,59,60], indicating that the roles of IRE1α-XBP1 on viral replication depend on viruses. 

IRE1-XBP1 pathway is required for, and regulates efficient protein folding, maturation, and degradation in response to ER stress [19]. We speculate that IRE1α RNase activity activates cellular protein degradation pathway (ERAD), and leads to the degradation of viral proteins, which is unfavourable to viral replication. This assumption was supported by our data that Tg-induced IRE1α RNase activity resulted in the inhibition of HSV-1 replication (Figure 2E,G). Results from an earlier study showed that PERK branch of UPR were activated by Tg through up-regulating phosphorylation of PERK [13]. PERK signaling regulates the suppression of translation initiation through phosphorylation of eIF2α to shutoff the cellular protein translation, and this response is used as an inhibitory mechanism on viral replication by the host. To facilitate viral replication, HSV-1 has evolved a countermeasure to avoid the detrimental effect of PERK through dephosphorylation of the eIF2α, mediated by a virus encoded protein γ_1_ 34.5 [61,62]. The ATF6 arm of UPR was also activated by Tg [29]. Researchers suggested that the activation of ATF6 and its transcriptional activation of chaperone-encoding genes might benefit the virus by assisting the folding of accumulated proteins and preventing protein aggregation. This idea was supported by observations showing that ATF6 signaling was stimulated by virus infections, such as HCV and African swine fever virus (ASFV), and beneficial to viral replication [11,63]. Therefore, we speculate that the Tg induced inhibition of viral replication was specifically mediated by its regulation of IRE1α branch, which was further supported by the observation that XBP1s over-expression also led to the viral inhibition (Figure 2I). The limited inhibitory effect of Tg-induced RNase activity of IRE1α (about 50% inhibition of HSV-1 replication (Figure 2E,G)) may reflect a viral countermeasure to suppress RNase activity of IRE1α, as an earlier report showed that IRE1-XBP1 signal was inhibited by HSV-1 UL41 protein [30]. We also observed that the transcriptions of XBP1s target genes were up-regulated by Tg, but this up-regulation was suppressed in HSV-1 infected cells (Figure 2J); however, detailed mechanisms of this feedback regulation need further investigation. In agreement with earlier studies that IRE1-XBP1 arm of UPR was inactivated or suppressed by HSV-1 infection [27,29,30], we further presented evidence that the activation of the RNase activity of IRE1α is detrimental to HSV-1 replication, and suppression of the RNase activity may serve as a mechanism for the virus to escape from host restriction. 

JNK pathway is activated under cellular stresses [64]. The protein kinases mitogen-activated protein kinase, kinase (MKK) 4 and MKK7 activate the JNK in response to pro-inflammatory cytokines or cellular stresses [65]. IRE1 signaling also modulates the JNK activation when UPR occurred [22]. Both JNK and p38 MAPK are activated by HSV infection [66]. However, evidence on how proximal signals are coupled to the activation of JNK kinase during HSV-1 infection is limited. Our data showed that HSV-1-stimulated JNK activation was repressed when IRE1α expression was down-modulated by RNA interference, indicating that IRE1α was one of the upstream regulators of the JNK cascade during HSV-1 infection (Figure 4E). We showed that IRE1α kinase activity positively regulated the HSV-1 replication (Figure 3), while IRE1α RNase activity negatively regulated viral replication (Figure 2E–I), suggesting that HSV-1-induced JNK cascade activation was mediated by IRE1α kinase activity. We further confirmed this result by over-expressing IRE1KA, or using the kinase inhibitor, both of which resulted in the reduced activation of JNK and viral replication. These data strongly suggest that IRE1α kinase activity is required for HSV-1 replication due to its stimulation of JNK kinase cascade in infected cells. Previous studies showed that HSV-1 viral proteins, such as ICP0, ICP27, or VP16, were correlated with JNK activation [32,66,67]. Since co-localizations of ICP0 or gD with IRE1α were observed in our preliminary study (data not shown), we speculate that the viral protein(s) might participate in the regulation of the IRE1α kinase activity to facilitate HSV-1 replication. Further investigation still need be done to unveil the detailed mechanisms.

In conclusion, our current study found that the activation of RNase activity of IRE1α has an inhibitory effect on HSV-1 replication, which might act as a cellular restriction to HSV-1 replication through activation of a downstream ER protein degradation pathway to limit viral protein production. But IRE1α kinase activity was helpful to HSV-1 replication. To counter the cellular restriction, HSV-1 inactivates the RNase activity, while activating the kinase activity of IRE1α through a yet unknown mechanism. This differential role of IRE1α activation on HSV-1 replication allows the virus to avoid detrimental consequence of UPR while exploiting the activation of JNK signaling pathway to facilitate its replication. It warrants further investigations to delineate the molecular mechanisms that dictate the elevation of kinase activities and the related viral factors.

## Supplementary Materials

The following are available online at www.mdpi.com/1999-4915/9/9/235/s1, Figure S1: The cytotoxic effect of Tg, STF-083010 and APY29 on HEC-1-A and Hela cells.

## References

[1] Cai W., Astor T.L., Liptak L.M., Cho C., Coen D.M., Schaffer P.A. (1993). The herpes simplex virus type 1 regulatory protein ICP0 enhances virus replication during acute infection and reactivation from latency. J. Virol..

[2] Rajcani J., Durmanova V. (2000). Early expression of herpes simplex virus (HSV) proteins and reactivation of latent infection. Folia Microbiol. (Praha).

[3] Birkmann A., Zimmermann H. (2016). HSV antivirals—Current and future treatment options. Curr. Opin. Virol..

[4] Kaufman R.J. (1999). Stress signaling from the lumen of the endoplasmic reticulum: Coordination of gene transcriptional and translational controls. Genes Dev..

[5] Ozcan U., Cao Q., Yilmaz E., Lee A.H., Iwakoshi N.N., Ozdelen E., Tuncman G., Gorgun C., Glimcher L.H., Hotamisligil G.S. (2004). Endoplasmic reticulum stress links obesity, insulin action, and type 2 diabetes. Science.

[6] Qu L., Huang S., Baltzis D., Rivas-Estilla A.M., Pluquet O., Hatzoglou M., Koumenis C., Taya Y., Yoshimura A., Koromilas A.E. (2004). Endoplasmic reticulum stress induces p53 cytoplasmic localization and prevents p53-dependent apoptosis by a pathway involving glycogen synthase kinase-3beta. Genes Dev..

[7] Bhattacharyya S., Sen U., Vrati S. (2014). Regulated IRE1-dependent decay pathway is activated during Japanese encephalitis virus-induced unfolded protein response and benefits viral replication. J. Gen. Virol..

[8] He B. (2006). Viruses, endoplasmic reticulum stress, and interferon responses. Cell Death Differ..

[9] Lin J.H., Li H., Yasumura D., Cohen H.R., Zhang C., Panning B., Shokat K.M., Lavail M.M., Walter P. (2007). IRE1 signaling affects cell fate during the unfolded protein response. Science.

[10] Chen L., Xu S., Liu L., Wen X., Xu Y., Chen J., Teng J. (2014). Cab45S inhibits the ER stress-induced IRE1-JNK pathway and apoptosis via GRP78/BiP. Cell Death Dis..

[11] Tardif K.D., Mori K., Siddiqui A. (2002). Hepatitis C virus subgenomic replicons induce endoplasmic reticulum stress activating an intracellular signaling pathway. J. Virol..

[12] Su H.L., Liao L.C., Lin Y.L. (2002). Japanese encephalitis virus infection initiates endoplasmic reticulum stress and an unfolded protein response. J. Virol..

[13] Cheng G., Feng Z., He B. (2005). Herpes simplex virus 1 infection activates the endoplasmic reticulum resident kinase PERK and mediates eIF-2alpha dephosphorylation by the gamma(1)34.5 protein. J. Virol..

[14] Tirasophon W., Welihinda A.A., Kaufman R.J. (1998). A stress response pathway from the endoplasmic reticulum to the nucleus requires a novel bifunctional protein kinase/endoribonuclease (ire1p) in mammalian cells. Genes Dev..

[15] Wang X.Z., Harding H.P., Zhang Y.H., Jolicoeur E.M., Kuroda M., Ron D. (1998). Cloning of mammalian ire1 reveals diversity in the ER stress responses. EMBO J..

[16] Credle J.J., Finer-Moore J.S., Papa F.R., Stroud R.M., Walter P. (2005). On the mechanism of sensing unfolded protein in the endoplasmic reticulum. Proc. Natl. Acad. Sci. USA.

[17] Bertolotti A., Zhang Y., Hendershot L.M., Harding H.P., Ron D. (2000). Dynamic interaction of BiP and ER stress transducers in the unfolded-protein response. Nat. Cell Biol..

[18] Ruegsegger U., Leber J.H., Walter P. (2001). Block of HAC1 mRNA translation by long-range base pairing is released by cytoplasmic splicing upon induction of the unfolded protein response. Cell.

[19] Lee A.H., Iwakoshi N.N., Glimcher L.H. (2003). XBP-1 regulates a subset of endoplasmic reticulum resident chaperone genes in the unfolded protein response. Mol. Cell. Biol..

[20] Hollien J., Weissman J. (2006). Decay of endoplasmic reticulum-localized mRNAs during the unfolded protein response. Science.

[21] Coelho D.S., Domingos P.M. (2014). Physiological roles of regulated ire1 dependent decay. Front. Genet..

[22] Urano F. (2000). Coupling of stress in the ER to activation of JNK protein kinases by transmembrane protein kinase IRE1. Science.

[23] Hassan I.H., Zhang M.S., Powers L.S., Shao J.Q., Baltrusaitis J., Rutkowski D.T., Legge K., Monick M.M. (2012). Influenza A viral replication is blocked by inhibition of the inositol-requiring enzyme 1 (IRE1) stress pathway. J. Biol. Chem..

[24] Bechill J., Chen Z., Brewer J.W., Baker S.C. (2008). Coronavirus infection modulates the unfolded protein response and mediates sustained translational repression. J. Virol..

[25] Tardif K.D., Mori K., Kaufman R.J., Siddiqui A. (2004). Hepatitis C virus suppresses the IRE1-XBP1 pathway of the unfolded protein response. J. Biol. Chem..

[26] Isler J.A., Skalet A.H., Alwine J.C. (2005). Human cytomegalovirus infection activates and regulates the unfolded protein response. J. Virol..

[27] Mulvey M., Arias C., Mohr I. (2007). Maintenance of endoplasmic reticulum (ER) homeostasis in herpes simplex virus type 1-infected cells through the association of a viral glycoprotein with PERK, a cellular ER stress sensor. J. Virol..

[28] Chou J., Chen J., Gross M., Roizman B. (1995). Association of a M(r) 90,000 phosphoprotein with protein kinase PKR in cells exhibiting enhanced phosphorylation of translation initiation factor eIF-2 alpha and premature shutoff of protein synthesis after infection with gamma (1)34.5-mutants of herpes simplex virus 1. Proc. Natl. Acad. Sci. USA.

[29] Burnett H.F., Audas T.E., Liang G., Lu R.R. (2012). Herpes simplex virus-1 disarms the unfolded protein response in the early stages of infection. Cell Stress Chaperones.

[30] Zhang P., Su C., Jiang Z., Zheng C., Sandri-Goldin R.M. (2017). Herpes simplex virus 1 UL41 protein suppresses the IRE1/XBP1 signal pathway of the unfolded protein response via its RNase activity. J. Virol..

[31] McLean T.I., Bachenheimer S.L. (1999). Activation of cJUN N-terminal kinase by herpes simplex virus type 1 enhances viral replication. J. Virol..

[32] Diao L., Zhang B., Xuan C., Sun S., Yang K., Tang Y., Qiao W., Chen Q., Geng Y., Wang C. (2005). Activation of c-Jun N-terminal kinase (JNK) pathway by HSV-1 immediate early protein ICP0. Exp. Cell Res..

[33] Flexner S. (1928). Contributions to the pathology of experimental virus encephalitis. IV. Recurring strains of herpes virus. J. Exp. Med..

[34] Qiu M., Chen Y., Chu Y., Song S., Yang N., Gao J., Wu Z. (2013). Zinc ionophores pyrithione inhibits herpes simplex virus replication through interfering with proteasome function and NF-kappaB activation. Antivir. Res..

[35] Qiu M., Chen Y., Cheng L., Chu Y., Song H.Y., Wu Z.W. (2013). Pyrrolidine dithiocarbamate inhibits herpes simplex virus 1 and 2 replication, and its activity may be mediated through dysregulation of the ubiquitin-proteasome system. J. Virol..

[36] Song S., Qiu M., Chu Y., Chen D., Wang X., Su A., Wu Z. (2014). Downregulation of cellular c-Jun N-terminal protein kinase and NF-kappaB activation by berberine may result in inhibition of herpes simplex virus replication. Antimicrob. Agents Chemother..

[37] McLean C., Erturk M., Jennings R., Challanain D.N., Minson A., Duncan I., Boursnell M., Inglis S. (1994). Protective vaccination against primary and recurrent disease caused by herpes simplex virus (HSV) type 2 using a genetically disabled HSV-1. J. Infect. Dis..

[38] Hirota M., Kitagaki M., Itagaki H., Aiba S. (2006). Quantitative measurement of spliced XBP1 mRNA as an indicator of endoplasmic reticulum stress. J. Toxicol. Sci..

[39] Yoshida H. (2007). Unconventional splicing of XBP-1 mRNA in the unfolded protein response. Antioxid. Redox Signal..

[40] Romero-Ramirez L., Cao H.B., Nelson D., Hammond E., Lee A.H., Yoshida H., Mori K., Glimcher L.H., Denko N.C., Giaccia A.J. (2004). XBP1 is essential for survival under hypoxic conditions and is required for tumor growth. Cancer Res..

[41] Back S.H., Lee K., Vink E., Kaufman R.J. (2006). Cytoplasmic IRE1alpha-mediated XBP1 mRNA splicing in the absence of nuclear processing and endoplasmic reticulum stress. J. Biol. Chem..

[42] Kijima Y., Ogunbunmi E., Fleischer S. (1991). Drug action of thapsigargin on the Ca^2+^ pump protein of sarcoplasmic reticulum. J. Biol. Chem..

[43] Yamamoto K., Yoshida H., Kokame K., Kaufman R.J., Mori K. (2004). Differential contributions of ATF6 and XBP1 to the activation of endoplasmic reticulum stress-responsive cis-acting elements ERSE, UPRE and ERSE-II. J. Biol. Chem..

[44] Saeed M., Suzuki R., Watanabe N., Masaki T., Tomonaga M., Muhammad A., Kato T., Matsuura Y., Watanabe H., Wakita T. (2011). Role of the endoplasmic reticulum-associated degradation (ERAD) pathway in degradation of hepatitis C virus envelope proteins and production of virus particles. J. Biol. Chem..

[45] Papandreou I., Denko N.C., Olson M., Van Melckebeke H., Lust S., Tam A., Solow-Cordero D.E., Bouley D.M., Offner F., Niwa M. (2011). Identification of an ire1alpha endonuclease specific inhibitor with cytotoxic activity against human multiple myeloma. Blood.

[46] Dicker I.B., Seetharam S. (1995). Herpes simplex type 1: LacZ recombinant viruses. I. Characterization and application to defining the mechanisms of action of known antiherpes agents. Antivir. Res..

[47] Zhu Q.C., Wang Y., Peng T. (2010). Herpes simplex virus (HSV) immediate-early (IE) promoter-directed reporter system for the screening of antiherpetics targeting the early stage of HSV infection. J. Biomol. Screen..

[48] Hosokawa N., Wada I., Hasegawa K., Yorihuzi T., Tremblay L.O., Herscovics A., Nagata K. (2001). A novel ER alpha-mannosidase-like protein accelerates er-associated degradation. EMBO Rep..

[49] Wang L., Perera B.G., Hari S.B., Bhhatarai B., Backes B.J., Seeliger M.A., Schurer S.C., Oakes S.A., Papa F.R., Maly D.J. (2012). Divergent allosteric control of the IRE1alpha endoribonuclease using kinase inhibitors. Nat. Chem. Biol..

[50] Korennykh A.V., Egea P.F., Korostelev A.A., Finer-Moore J., Zhang C., Shokat K.M., Stroud R.M., Walter P. (2009). The unfolded protein response signals through high-order assembly of IRE1. Nature.

[51] Cross B.C., Bond P.J., Sadowski P.G., Jha B.K., Zak J., Goodman J.M., Silverman R.H., Neubert T.A., Baxendale I.R., Ron D. (2012). The molecular basis for selective inhibition of unconventional mRNA splicing by an IRE1-binding small molecule. Proc. Natl. Acad. Sci. USA.

[52] Han D., Lerner A.G., Vande Walle L., Upton J.P., Xu W., Hagen A., Backes B.J., Oakes S.A., Papa F.R. (2009). IRE1alpha kinase activation modes control alternate endoribonuclease outputs to determine divergent cell fates. Cell.

[53] Lipson K.L., Fonseca S.G., Ishigaki S., Nguyen L.X., Foss E., Bortell R., Rossini A.A., Urano F. (2006). Regulation of insulin biosynthesis in pancreatic beta cells by an endoplasmic reticulum-resident protein kinase IRE1. Cell Metab..

[54] Chen D., Su A., Fu Y., Wang X., Lv X., Xu W., Xu S., Wang H., Wu Z. (2015). Harmine blocks herpes simplex virus infection through downregulating cellular NF-kappaB and MAPK pathways induced by oxidative stress. Antivir. Res..

[55] Tam A.B., Koong A.C., Niwa M. (2014). IRE1 has distinct catalytic mechanisms for XBP1/HAC1 splicing and RIDD. Cell Rep..

[56] Maurel M., Chevet E., Tavernier J., Gerlo S. (2014). Getting RIDD of RNA: IRE1 in cell fate regulation. Trends Biochem. Sci..

[57] Smith J.A., Schmechel S.C., Raghavan A., Abelson M., Reilly C., Katze M.G., Kaufman R.J., Bohjanen P.R., Schiff L.A. (2006). Reovirus induces and benefits from an integrated cellular stress response. J. Virol..

[58] Li B., Gao B., Ye L., Han X., Wang W., Kong L., Fang X., Zeng Y., Zheng H., Li S. (2007). Hepatitis B virus x protein (HBX) activates ATF6 and IRE1-XBP1 pathways of unfolded protein response. Virus Res..

[59] Yu C.Y., Hsu Y., Liao C.L., Lin Y.L. (2006). Flavivirus infection activates the XBP1 pathway of the unfolded protein response to cope with endoplasmic reticulum stress. J. Virol..

[60] Lee D.Y., Sugden B. (2008). The LMP1 oncogene of EBV activates PERK and the unfolded protein response to drive its own synthesis. Blood.

[61] Mulvey M., Arias C., Mohr I. (2006). Resistance of mRNA translation to acute endoplasmic reticulum stress-inducing agents in herpes simplex virus type 1-infected cells requires multiple virus-encoded functions. J. Virol..

[62] Cerveny M., Hessefort S., Yang K., Cheng G., Gross M., He B. (2003). Amino acid substitutions in the effector domain of the gamma(1)34.5 protein of herpes simplex virus 1 have differential effects on viral response to interferon-α. Virology.

[63] Galindo I., Hernáez B., Muñoz-Moreno R., Cuesta-Geijo M.A., Dalmau-Mena I., Alonso C. (2012). The ATF6 branch of unfolded protein response and apoptosis are activated to promote african swine fever virus infection. Cell Death Dis..

[64] Bogoyevitch M.A., Ngoei K.R., Zhao T.T., Yeap Y.Y., Ng D.C. (2010). c-Jun N-terminal kinase (JNK) signaling: Recent advances and challenges. Biochim. Biophys. Acta.

[65] Fleming Y., Armstrong C.G., Morrice N., Paterson A., Goedert M., Cohen P. (2000). Synergistic activation of stress-activated protein kinase 1/c-Jun N-terminal kinase (SAPK1/JNK) isoforms by mitogen-activated protein kinase kinase 4 (MKK4) and MKK7. Biochem. J..

[66] Zachos G., Clements B., Conner J. (1999). Herpes simplex virus type 1 infection stimulates p38/c-Jun N-terminal mitogen-activated protein kinase pathways and activates transcription factor AP-1. J. Biol. Chem..

[67] Hargett D., McLean T., Bachenheimer S.L. (2005). Herpes simplex virus ICP27 activation of stress kinases JNK and p38. J. Virol..

