# Supplementary material for: Opposite Roles of RNase and Kinase Activities of Inositol-Requiring Enzyme 1 (IRE1) on HSV-1 Replication
Source: Viruses. 2017 Aug 23;9(9):235. doi: 10.3390/v9090235 (PMC5618002; doi:10.3390/v9090235)
Supplement: Supplementary file 1 [file viruses-09-00235-s001.pdf]

Figure S1

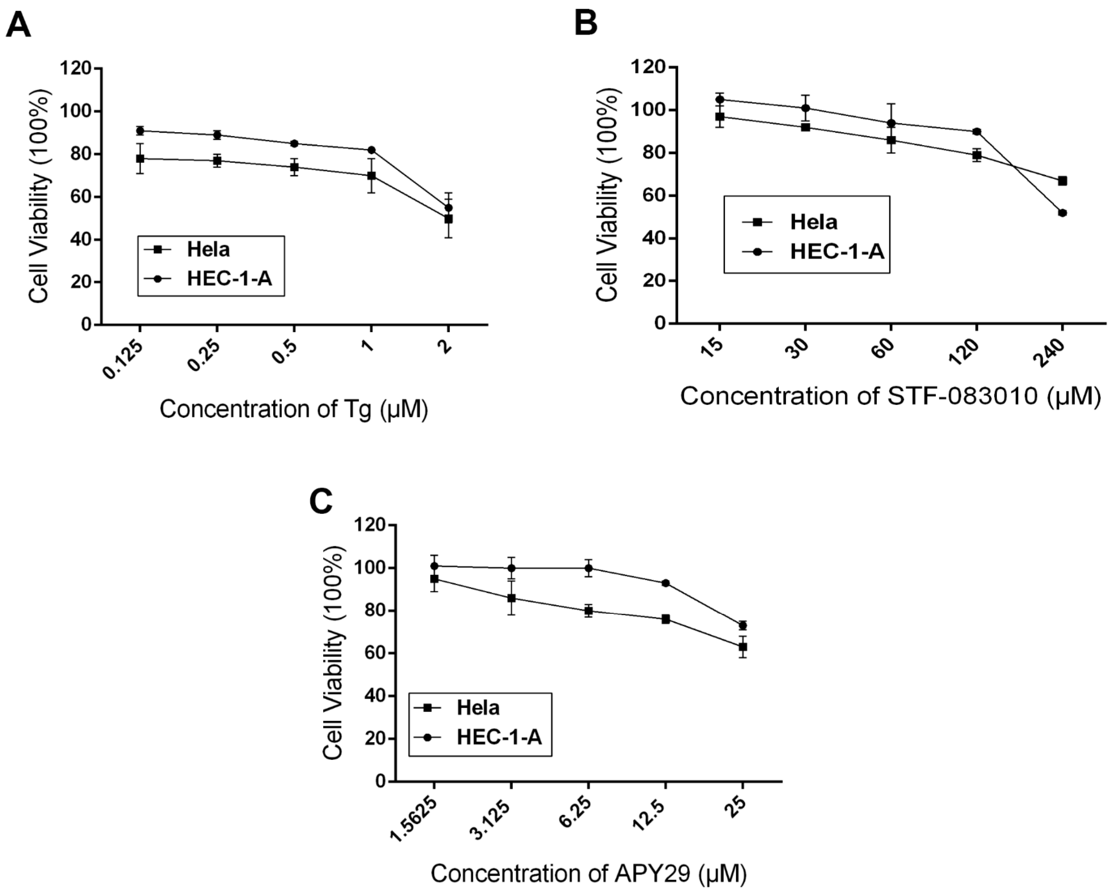

Figure S1: The in vitro cytotoxicity of Tg (A), STF-083010 (B) and APY29 (C) was measured using a commercial CCK-8 kit (Dojindo, Kumamoto, Japan) via colorimetric method according to the manufacturer's instructions. Briefly, 2×104 cells per well were seeded into 96-well plates and cultured for 24 hrs before serial concentrations of drugs were added in triplicate. After 24 hrs, 10μl CCK-8 reagent was dispensed into each well, and the plates were incubated at 37°C for 3 hrs. Absorbance at 450nm was measured using a TECAN Infinite M200 microplate reader (Männedorf, Switzerland). Cell viability was plotted as the percent viable cells of the mock-treated control cells. All experiments were performed three times. The representative results were shown. Data are mean values (±SD) of triplicate determinations.
